# Demonstration of Binding Induced Structural Plasticity in a SH2 Domain

**DOI:** 10.3389/fmolb.2020.00089

**Published:** 2020-05-12

**Authors:** Lorenzo Visconti, Angelo Toto, James A. Jarvis, Francesca Troilo, Francesca Malagrinò, Alfonso De Simone, Stefano Gianni

**Affiliations:** ^1^Istituto Pasteur – Fondazione Cenci Bolognetti, Dipartimento di Scienze Biochimiche “A. Rossi Fanelli” and Istituto di Biologia e Patologia Molecolari del CNR, Sapienza Università di Roma, Rome, Italy; ^2^Department of Life Sciences, Imperial College London, London, United Kingdom

**Keywords:** allosteric network, skinetics, mutagenesis, peptide binding, NMR

## Abstract

SH2 domains are common protein interaction domains able to recognize short aminoacidic sequences presenting a phosphorylated tyrosine (pY). In spite of their fundamental importance for cell physiology there is a lack of information about the mechanism by which these domains recognize and bind their natural ligands. The N-terminal SH2 (N-SH2) domain of PI3K mediates the interaction with different scaffolding proteins and is known to recognize a specific pY-X-X-M consensus sequence. These interactions are at the cross roads of different molecular pathways and play a key role for cell development and division. By combining mutagenesis, chemical kinetics and NMR, here we provide a complete characterization of the interaction between N-SH2 and a peptide mimicking the scaffolding protein Gab2. Our results highlight that N-SH2 is characterized by a remarkable structural plasticity, with the binding reaction being mediated by a diffused structural region and not solely by the residues located in the binding pocket. Furthermore, the analysis of kinetic data allow us to pinpoint an allosteric network involving residues far from the binding pocket involved in specificity. Results are discussed on the light of previous works on the binding properties of SH2 domains.

## Introduction

The interaction between proteins plays a paramount role in biological functions. Frequently, protein-protein recognition is mediated by specific domains such as SH2, SH3, WW, or PDZ domains (Macias et al., 1996; Pawson and Scott, 1997; Bhattacharyya et al., 2006; Teyra et al., 2012; Kurochkina and Guha, 2013). Whilst these domains are typically highly conserved and often very abundant in the cellular environment, they are nevertheless specific, such as cross-reactivity with non-desired partners is minimized (Zarrinpar et al., 2003). It has been observed that PDZ and SH3 domains control their ability to recognize specific consensus sequences through dynamic and energetic allosteric mechanisms (Lockless and Ranganathan, 1999; Fuentes et al., 2006; Gianni et al., 2011; Kumawat and Chakrabarty, 2017; Malagrinò et al., 2019). These mechanisms overcome the classical paradigm of allostery, which is based on major protein structural and conformational changes, and are characterized by fine rearrangement in the dynamic of the system (Kern and Zuiderweg, 2003; Motlagh et al., 2014). This behavior has been demonstrated for different protein-protein interaction domains. However, despite their critical roles in many important biological pathways, little information is currently available on Src Homology 2 (SH2) domains, for which only little evidence of interdomain allosteric regulations has been reported (Tsutsui et al., 2016; Gangopadhyay et al., 2020).

SH2 domains represent a classical example of modular protein interaction domains. SH2 domains are present in a wide range of proteins, including kinases, adaptors, phosphatases and other signaling molecules (Songyang et al., 1993; Payne et al., 1994), counting for more than 110 different proteins encoded by human genome. From a structural point of view, the SH2 domains are composed of about 100 amino acids presenting a conserved architecture comprising three to seven anti-parallel β strands organized in a β sheet, flanked by two α-helices (Waksman et al., 1992, 1993). SH2 domains from different proteins are known to recognize specific consensus sequences containing a phosphorylated tyrosine (pY) (Payne et al., 1994). In addition to the phospho-tyrosine, each SH2 domain recognizes several additional flanking residues, generally three to five amino acids C-terminal to pY (Schaffhausen, 1995). Many signaling pathways require the action of SH2 domains to control the colocalization of a variety of proteins within the signaling cascade, the binding events mediated by these domains allowing a correct signal transduction (Koch et al., 1991; Neel et al., 2003). For these reasons, mutations that affect the binding properties of the SH2 domains lead to an abnormal regulation of important signaling pathways, causing the misregulation of key physiological processes in the cell and consequently to the development of a number of diseases (Tartaglia et al., 2001; Lappalainen et al., 2008).

PI3K proteins interact with various RTKs involved in an ample range of signaling processes (Katso et al., 2001; Engelman et al., 2006). The activation of PI3K requires the interaction of its two SH2 domains with the binding partners, thus driving the phosphorylation of phosphatidylinositols at their 3′ position. Both the N-terminal and the C-terminal SH2 domains (N-SH2 and C-SH2 respectively) recognize the consensus pY-X-X-M sequence of the target (Waksman et al., 1992; Songyang et al., 1993). The interaction between PI3K and their ligands is very often mediated by scaffolding proteins like, for example, Gab1, Gab2, IRS-1, FRS2, that act as platforms recruiting several transcription factors and allowing the correct formation of the molecular machinery.

The Grb2-associated binding protein (Gab2) is composed by an N-terminal folded PH domain and C-terminal unfolded region where the binding sites for other signaling proteins are situated. Because of its key role as scaffolding protein and in the binding with several transcription factors, Gab2 is implicated in several cancers of both solid and hematological origin (Ke et al., 2007; Ding et al., 2015). In particular Gab2 is overexpressed in breast (Bentires-Alj et al., 2006), gastric (Lee et al., 2007) and lung (Xu et al., 2011) cancers, while its expression in healthy mature cells is relatively suppressed.

Here we provide, through a combination of stopped-flow and NMR experiments, a detailed characterization of the interaction between the N-terminal SH2 domain of PI3K and a 13-residue peptide mimicking Gab2, in its wild-type form and a variant M→A affecting the specific consensus pY-X-X-M sequence. Whilst the structural features of the N-SH2 has been previously described both in the free and in the bound state with other partners (Nolte et al., 1996), the mechanism of interaction of this protein with its natural ligands has never been characterized. Our data show that N-SH2 displays an unexpected structural plasticity, which does not reflect as major structural changes upon binding, but as a finely regulated rearrangement of the entire domain. Binding capabilities of N-SH2 are not solely mediated by its binding pocket but are modulated diffusely within its structure. In fact, by combining site-directed mutagenesis with kinetic and NMR experiments we provide compelling evidence highlighting a remarkable structural malleability of N-SH2 domain. The analysis of kinetic data allowed us to depict an allosteric network involving residues that are not located in the binding site but nevertheless appear to play a role in the recognition of the M residue, which is part of the consensus sequence of N-SH2. These results provide the first description of an allosteric network in the binding reaction of a SH2 domain.

## Materials and Methods

### Protein Expression and Purification

N-SH2 wild-type and all the site-directed variants were expressed and purified as described previously (Visconti et al., 2019). All the mutants were obtained using a QuickChange Lightning Site-Directed Mutagenesis kit (Agilent technologies), accordingly to manufacturer instructions. Peptides with sequence TNSEDNpYVPMNPG mimicking wild-type Gab2 and TNSEDNpYVPANPG mimicking the M457A variant of Gab2 were purchased from GenScript Biotech Corporation.

### Stopped-Flow Experiments

Binding kinetics experiments were performed using a single-mixing SX-18 stopped-flow instrument (Applied Photophysics); all binding experiments were conducted in 50 mM Hepes pH 7.4 300 mM NaCl at 10°C. Excitation was at 280 nM and emission was collected using a 475 nM cut-off glass filter. Pseudo-first order binding experiments were performed mixing a constant concentration of dansylated Gab2_448__–__460_ and Gab2_448__–__460_ M457A (1 μM) vs. N-SH2 wt and its mutants at concentrations ranging from 4 to 12 μM. For each N-SH2 concentration, usually 5 individual traces were averaged and in all cases the fluorescence time courses obtained was satisfactorily fitted by using a single exponential equation.

### NMR Experiments

Standard triple resonance experiments [CBCA(CO)NH, HNCACB, HNCO, HN(CA)CO spectra] and HSQC were recorded at 10°C on Bruker spectrometers operating at ^1^H frequencies of 600 MHz. Solution NMR experiments were carried out in buffer 50 mM Hepes pH 7.4 300 mM NaCl using a 100 μM sample of labeled N-SH2. For the full assignment of the bound N-SH2 we performed titrations of ^1^H-^15^N HSQC spectra of a 100 μM sample of N-SH2 with both Gab2_448__–__460_ and Gab2_448__–__460_ M457A, which were recorded using progressive concentration of the ligand until 1:1 molar ratio.

## Results

### Stopped-Flow Binding Kinetics

**FIGURE 1:**
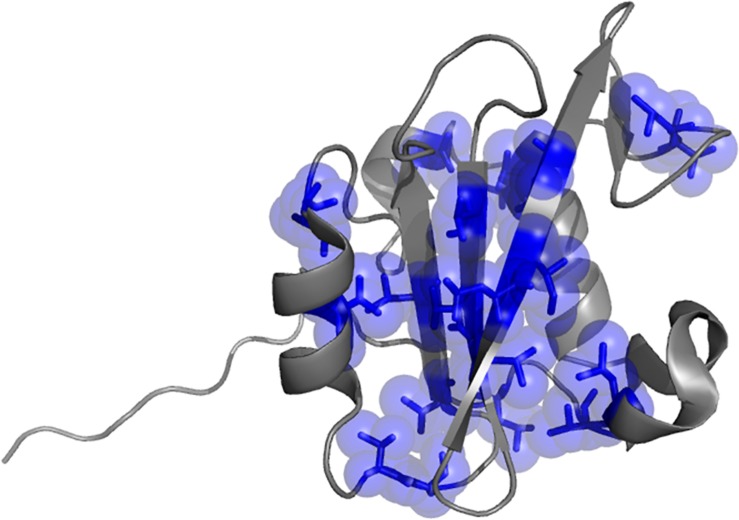
Three dimensional structure of the N-terminal SH2 domain of PI3K (PDB: 2IUH). Selected positions mutated for the kinetic analysis are highlighted as blue sticks and spheres.

**FIGURE 2:**
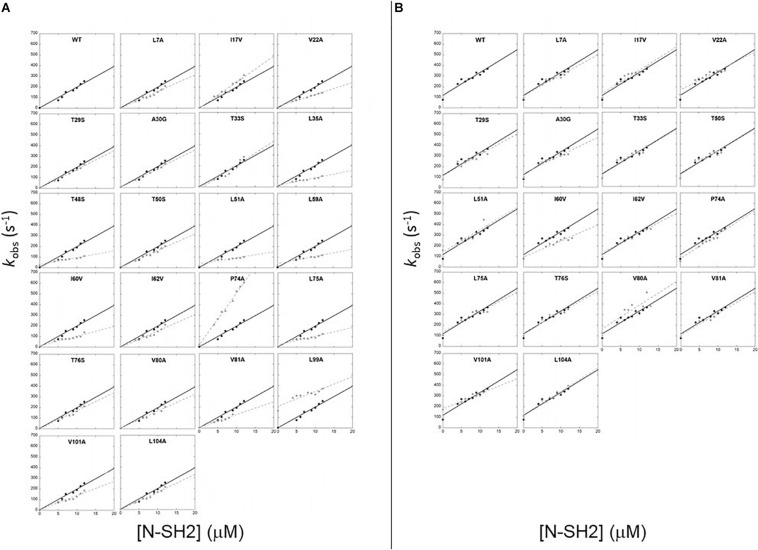
Pseudo first order binding experiments between N-SH2 in its wt form (black circles) and its variants (gray circles) vs Gab2_448__–__460_ **(A)** and Gab2_448__–__460_ M457A **(B)**. Black continuous lines for N-SH2 wt and gray broken lines for N-SH2 variants represent the best fit to a linear equation (see text for details).

One of the aims of this study is to depict the molecular details of the interaction between the N-SH2 domain of PI3K and Gab2. A powerful strategy to achieve this goal relies on perturbing the system by producing conservative site-directed variants while monitoring the effect of mutations on the association and dissociation rate constants between the interacting partners (*k*_on_ and *k*_off_ respectively). Thus, we designed and successfully produced 21 site-directed variants of the N-SH2 domain and challenged them vs. a peptide mimicking the region of Gab2 ranging from residue 448–460 (Gab2_448__–__460_), containing the pY-X-X-M motif that is specifically recognized by the N-SH2 domain (Waksman et al., 1992; Songyang et al., 1993). The choise of mutations was designed in analogy to phi-value analysis methodology in protein folding studies (Fersht and Sato, 2004). Positions mutated are showed in Figure 1. To monitor binding by fluorescence, a dansyl group was added at the N-terminus of the peptide. The binding reaction was monitored by stopped-flow experiments following the change in FRET signal, taking advantage of the two Trp residues on the N-SH2 domain in positions 12 and 14 as fluorescence donor and the dansyl group on Gab2_448__–__460_ as acceptor. The experiments were performed in pseudo-first order conditions with Gab2_448__–__460_ at fixed concentration of 1 μM rapidly mixed vs. large excess of N-SH2 wt and its variants, ranging from 4 to 14 μM, in buffer Hepes 50 mM, NaCl 300 mM, pH 7.4, at 10°C. For all the experiments observed time courses were found to be consistent with a single exponential equation. The dependence of observed rate constants *k*_obs_ as a function of the concentration of N-SH2 was fitted with a linear equation, suggesting that the binding reaction can be described by a simple two-state mechanism

$$k_{o\,b\,s}=\left[N\,S\,H\,2\right]\,k_{o\,n}+k_{o\,f\,f}$$

with *k*_on_ being the slope of the line and *k*_off_ the intercept with the y-axis. Since indirect calculation of *k*_off_ obtained by extrapolation is associated with high experimental error, we performed displacement experiments to directly measure *k*_off_ by challenging a pre-incubated complex of dansylated Gab2_448__–__460_ and N-SH2 domain both at the concentration of 1 μM vs. a large excess of non-dansylated Gab2_448__–__460_ (50 μM). The pseudo-first order plots of *k*_obs_ vs. the concentration of wild-type N-SH2 and its variants are shown in Figure 2A. Kinetic parameters extracted from data together with the calculated changes in activation and equilibrium free energies are reported in Table 1. Analysis of kinetic data highlights that T48S, L51A, and L59A variations cause a detectable decrease of the affinity of the domain for Gab2_448__–__460_ of ∼10-fold, affecting both the *k*_on_ and *k*_off_. Furthermore, L99A mutation displays a strong destabilization effect on the complex, with an increase of dissociation equilibrium rate constant, K_D_, of ~100-fold, mainly caused by a high increase of *k*_off_. Since L99 residue is part of the binding pocket, this dramatic effect on the binding affinity is expected.

**TABLE 1:** Kinetic and thermodynamic parameters obtained from the analysis of the pseudo-first order binding experiments between site-directed variants of N-SH2 and Gab2_448__–__460_ wild-type and Gab2_448__–__460_ M457A.

	Gab2_448__–__460_				Gab2_448__–__460_ M457A				
N-SH2 variant	*k*_on_ (μM^–1^ s^–1^)	*k*_off_ (s^–1^)	K_D_ (μM)	ΔΔG_eq_ (kcal mol^–1^)	*k*_on_ (μM^–1^ s^–1^)	*k*_off_ (s^–1^)	K_D_ (μM)	ΔΔG_eq_ (kcal mol^–1^)	ΔΔΔ G
WT	19.5 ± 1.0	3.9 ± 0.2	0.2 ± 0.1		21.5 ± 2.4	80 ± 4	3.7 ± 0.4		
L7A	15.7 ± 1.2	4.0 ± 0.2	0.3 ± 0.1	0.15 ± 0.03	19.3 ± 2.3	90 ± 4	4.6 ± 0.6	0.12 ± 0.01	**
I17V	24.4 ± 1.7	4.5 ± 0.2	0.2 ± 0.1	−0.05 ± 0.02	22.0 ± 2.3	90 ± 5	4.3 ± 0.5	0.09 ± 0.01	**
V22A	11.2 ± 0.4	7.4 ± 0.4	0.7 ± 0.1	0.68 ± 0.04	17.7 ± 2.6	170 ± 9	9.7 ± 1.5	0.55 ± 0.05	**
T29S	17.5 ± 0.5	4.3 ± 0.2	0.3 ± 0.1	0.13 ± 0.02	19.7 ± 2.7	80 ± 4	4.0 ± 0.6	0.05 ± 0.01	**
A30G	29.2 ± 2.0	3.7 ± 0.2	0.1 ± 0.1	−0.25 ± 0.02	17.1 ± 2.2	90 ± 4	5.1 ± 0.7	0.19 ± 0.10	**
T33S	22.6 ± 2.4	3.6 ± 0.2	0.2 ± 0.1	−0.12 ± 0.02	20.6 ± 2.9	80 ± 4	3.8 ± 0.6	0.02 ± 0.01	**
L35A	7.0 ± 0.6	6.7 ± 0.3	1.0 ± 0.1	0.88 ± 0.09	*	*	*	*	*
T48S	6.4 ± 0.5	23 ± 1	3.7 ± 0.1	1.64 ± 0.36	*	*	*	*	*
T50S	15.6 ± 0.8	9.3 ± 0.5	0.6 ± 0.1	0.62 ± 0.05	20.2 ± 2.0	140 ± 7	6.8 ± 0.8	0.35 ± 0.10	**
L51A	5.6 ± 1.1	18 ± 1	3.2 ± 0.2	1.56 ± 0.64	21.2 ± 3.3	160 ± 8	7.6 ± 1.2	0.41 ± 0.10	1.16 ± 0.42
L59A	6.7 ± 1.0	21 ± 1	3.2 ± 0.2	1.57 ± 0.52	*	*	*	*	*
I60V	9.1 ± 1.1	6.9 ± 0.3	0.8 ± 0.1	0.76 ± 0.10	15.2 ± 1.5	80 ± 4	5.0 ± 0.6	0.18 ± 0.02	0.58 ± 0.16
I62V	15.5 ± 1.4	4.5 ± 0.2	0.3 ± 0.1	0.21 ± 0.03	18.4 ± 1.5	120 ± 6	6.7 ± 0.6	0.34 ± 0.10	**
P74A	48.7 ± 1.5	27 ± 1	0.6 ± 0.1	0.59 ± 0.04	22.5 ± 1.9	60 ± 3	2.6 ± 0.3	−0.20 ± 0.02	0.79 ± 0.12
L75A	8.1 ± 1.1	6.1 ± 0.3	0.8 ± 0.1	0.76 ± 0.11	18.8 ± 3.2	110 ± 5	5.7 ± 1.0	0.25 ± 0.03	0.51 ± 0.17
T76S	16.9 ± 1.3	5.3 ± 0.3	0.3 ± 0.1	0.26 ± 0.03	19.6 ± 2.6	100 ± 5	5.0 ± 0.7	0.17 ± 0.02	**
V80A	16.0 ± 1.2	4.4 ± 0.2	0.3 ± 0.1	0.19 ± 0.03	22.3 ± 5.4	130 ± 6	5.6 ± 1.4	0.24 ± 0.02	**
V81A	12.0 ± 1.4	4.5 ± 0.2	0.4 ± 0.1	0.37 ± 0.05	20.5 ± 3.8	90 ± 5	4.5 ± 0.9	0.11 ± 0.01	**
L99A	8.2 ± 1.7	160 ± 10	20 ± 0.2	2.59 ± 0.30	*	*	*	*	*
V101A	13.4 ± 0.4	7.0 ± 0.4	0.5 ± 0.1	0.55 ± 0.06	14.2 ± 1.7	170 ± 9	12 ± 1	0.68 ± 0.10	**
L104A	16.5 ± 0.2	3.5 ± 0.2	0.2 ± 0.1	0.05 ± 0.02	22.1 ± 1.4	110 ± 6	5.2 ± 0.4	0.19 ± 0.02	**

*Binding was abolished by this mutation. **The absolute value of calculated ΔΔΔG was below 0.4 kcal mol^–1^ and was excluded from analysis.

SH2 domains bind ligands with a phosphorylated tyrosine in their sequence. The N-SH2 domain of PI3K is known to recognize a specific consensus sequences that present a methionine in position +3 in respect to the phosphotyrosine (M457 in Gab2). To investigate the details of the role of M457 of Gab2 in the binding reaction we conducted kinetic binding experiments between Gab2_448__–__460_ M457A and all the site-directed variants of N-SH2 domains, under the same experimental conditions that were used for Gab2_448__–__460_. In analogy to what observed with the wild-type peptide, the fluorescence change upon binding followed a single exponential decay, and dependences of *k*_obs_ vs. the concentration of N-SH2 were fitted with a linear equation (Figure 2B). Kinetic data obtained from binding and displacement experiments are reported in Table 1. Our data show that M457 is not essential for binding, the K_D_ obtained for the complex with N-SH2 wt being 3.7 ± 0.4 μM. The analysis of microscopic association and dissociation rate contants highlights that *k*_on_ is comparable with the one calculated for the binding with Gab2_448__–__460_. However, a clear increase in *k*_off_ is reported for the binding with N-SH2 wt (Gab2_448__–__460_ *k*_off_ = 3.9 ± 0.2 s^–1^; Gab2_448__–__460_ M457A *k*_off_ = 80 ± 4 s^–1^) suggesting that M457 has a role in the dissociation of Gab2 with N-SH2, rather than in the recognition event. Interestingly, the *k*_on_ values obtained for the binding of Gab2_448__–__460_ M457A with all N-SH2 variants appear to be mostly unaffected by mutations occurring in N-SH2, whilst a more pronounced effect on the association rate constant is appreciable for the binding with wild type Gab2_448__–__460_. This difference may suggest that M457 may be energetically connected with the residues on N-SH2 displaying a different behavior, possibly highlighting a complex role in the binding reaction with the N-SH2 and, therefore, demanding further investigations.

### LFER Analysis and Characterization of TS of the Binding Reaction

**FIGURE 3:**
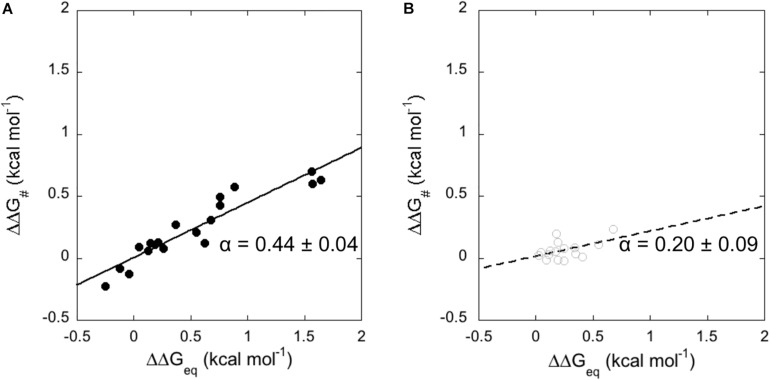
Linear Free Energy Relationship plots (LFER) obtained from the analysis of kinetic data for the binding of N-SH2 domain with Gab2_448__–__460_ (**A** – black full circles and black line) and with Gab2_448__–__460_ M457A (**B** – gray empty circles and gray broken line). Lines represent the best fit to a linear equation.

An essential element to characterize the mechanism of the binding reaction of a domain is the analysis of the transition state (TS) of the reaction. In this context, the study of the correlation between the free energy of the activation barrier to that of the ground states, classically denoted as linear free energy relationship plot (LFER plot) (Leffler, 1953), is a widely used strategy in organic chemistry to analyse reactions involving formation of covalent bonds, as well as in enzymology (Toney and Kirsch, 1989), protein folding (Fersht, 2004), and binding studies (Eaton et al., 1991). LFER analysis correlates the changes in free energy of the transition state to the changes in equilibrium free energies; the slope of the correlation being denoted as α representing the position of the transition state along the reaction coordinate. The mutational analysis performed in Table 1, allowed us to apply a LFER analysis on the interaction of N-SH2 with Gab2_448__–__460_ and Gab2_448__–__460_ M457A (Figure 3). Interestingly, in both cases, the measured data return a linear LFER plot. In analogy to what previously discussed in protein folding (Fersht, 2004), this finding is a hallmark of co-operativity and suggests that not only the residues located in the binding pocket, but all the probed residues taken are involved in the binding of the ligand.

It is of interest to compare the dependence of the plots measured for wild-type Gab2_448__–__460_ and Gab2_448__–__460_ M457A. In fact, whilst the apparent α value for the TS of N-SH2 upon binding with Gab2_448__–__460_ is 0.44 ± 0.04, in the case of Gab2_448__–__460_ M457A we measured a value of 0.20 ± 0.09. This finding would suggest that the M457A mutation may influence the structure of the transition state of N-SH2 in the binding reaction, suggesting that this protein may be characterized by a remarkable structural malleability that is dictated by the ligand. Because these differences appear however just above the limit of the experimental detection, to further validate this intriguing scenario we resorted to perform NMR experiments with the two different ligands.

### NMR

**FIGURE 4:**
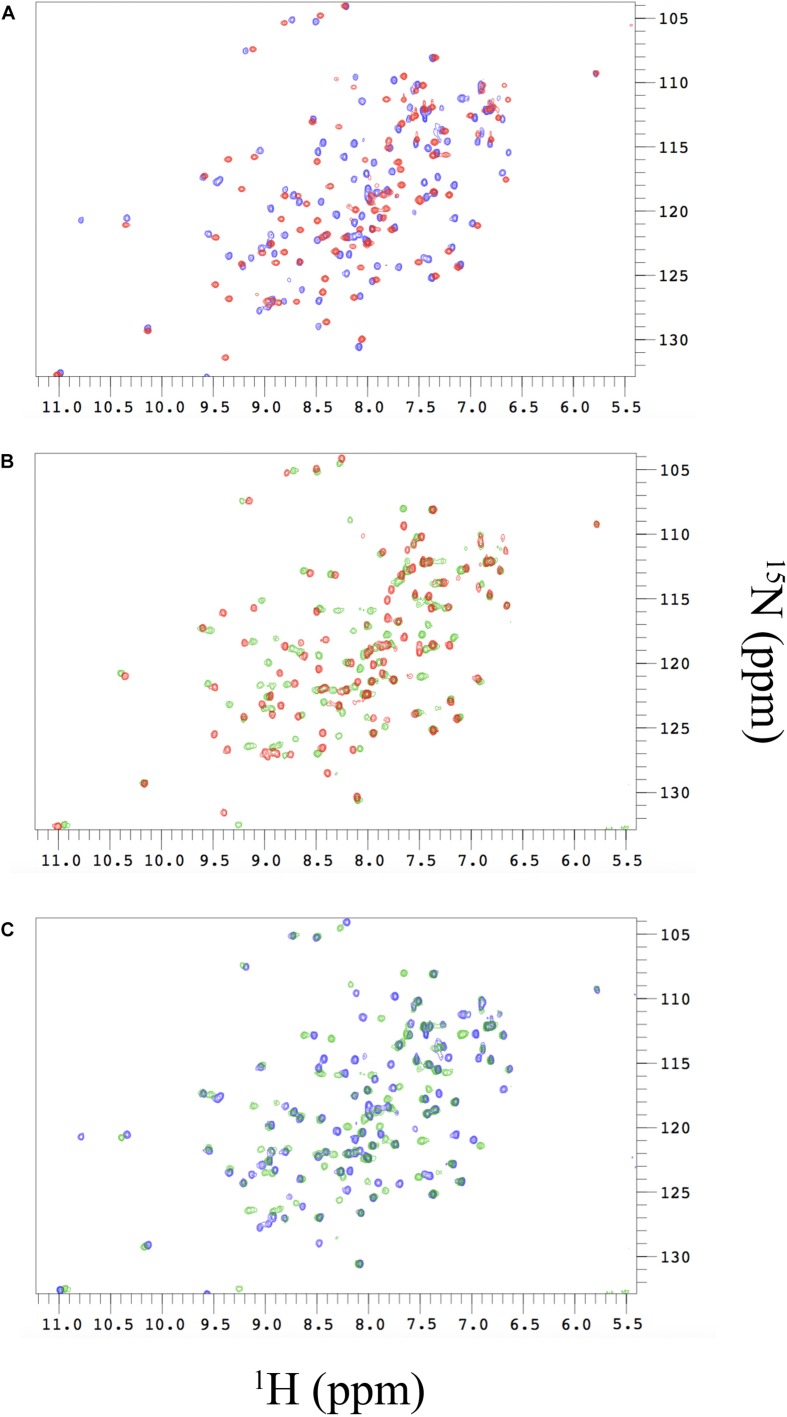
**(A)** Changes in the ^1^H-^15^N-HSQC spectra of free N-SH2 (red peaks) and N-SH2 in complex with Gab2_448__–__460_ (blue peaks). **(B)** Changes in the ^1^H-^15^N-HSQC spectra of free N-SH2 (red peaks) and N-SH2 in complex with Gab2_448__–__460_ M457A (green peaks). **(C)** Comparison of ^1^H-^15^N-HSQC spectra of the bound states of N-SH2 with the two variants of Gab2 peptide. The analysis reveals that the M457A mutation in the ligand modulates the plasticity of N-SH2 in the complex.

We then probed at an atomic resolution the structural malleability of N-SH2 using nuclear magnetic resonance (NMR). The sharp and well-dispersed backbone amide resonances in the ^1^H-^15^N HSQC of N-SH2 in the free state (Figure 4A, in red) indicate that the protein is well folded under the experimental conditions employed *in plastico*. To obtain residue-specific information from these spectra, we assigned the resonances of the protein backbone using a combination of 3D spectra, including CBCA(CO)NH, HNCACB, HNCO, HN(CA)CO, which were analyzed using a computer-aided procedure as described in Fusco et al. (2012). The assigned ^1^H-^15^N HSQC was employed to map the conformational changes and interaction surfaces of ^15^N labeled N-SH2 upon binding with increasing amounts of unlabeled Gab2_448__–__460_ or Gab2_448__–__460_ M457A in a titration experiment.

The resulting Chemical Shift Perturbation (CSP) is an established method to study protein-protein interactions, as it enables following the perturbations on the chemical environment of single residues upon ligand binding. Since changes in chemical shifts are very sensitive to structural changes, comparison of HSQC spectra of free and bound state of a protein may reveal important information about the residues involved in the process. In fact, whilst major changes are expected for residues that are in the binding interface, conformational changes occurring on distal residues, not directly involved in binding, may be diagnostic for allosteric communication in the protein.

The comparison between the HSQC of the N-SH2 in the free and in the bound state with Gab2_448__–__460_ shows marked chemical shift differences, indicating significant perturbation of the conformational properties of N-SH2 in the free and in the bound state (Figure 4A). Analogous results have been obtained for the binding of Gab2_448__–__460_ M457A (Figure 4B) with major chemical shift changes observed as a result of this interaction. CSP analysis clearly indicates the nature of the cooperative mechanism of binding of N-SH2 domain with Gab2_448__–__460_ and Gab2_448__–__460_ M457A. This result provides further evidence that the LFER linearity is due to a global structural perturbation of the protein domain upon binding. Interestingly, the comparison of the peak shifts in the bound states with Gab2_448__–__460_ and Gab2_448__–__460_ M457A evidences that the mutation occurring in the ligand affects the plasticity of N-SH2 in the complex (Figure 4C). Our results suggest that the interaction of M457 with the binding site of N-SH2 generates a structural response in distal residues of the protein. This scenario implies the presence of an allosteric network in N-SH2 that finely regulates the binding properties and conformations of SH2.

### Double Mutant Cycles

Protein allostery may be defined as the regulation of a given protein at a site other than the active (or binding) site. Allosteric regulation can be cryptic and escape characterization, occurring in the absence of major conformational changes and involving thermodynamic interactions of single distal residues. A powerful methodology to quantitatively calculate the interaction involved in such elusive allosteric regulations is to measure the energetic coupling between the ligand and residues that are not directly located in the binding pocket.

Double mutant cycle is a very effective strategy to infer energetic coupling and is based on the synergic employment of mutagenesis and binding experiments (Horovitz and Fersht, 1990). Thus, to investigate the selectivity of N-SH2 domain, we resorted to investigate the energetic coupling between its residues and position M457 of Gab2, which represents a key residue in driving the selectivity of the domain. In analogy to previous work on other domains (Chi et al., 2008; Gianni et al., 2011), coupling free energies were calculated by comparing the changes in free energy obtained for the N-SH2 variants when binding to Gab2_448__–__460_ to those obtained when binding Gab2_448__–__460_ M457A, following the formalism:

$$\Delta \,\Delta \,\Delta \,G=\Delta \,\Delta \,G_{e\,q\,G\,a\,b\,2\,w\,t}^{m\,u\,t-w\,t}-\Delta \,\Delta \,G_{e\,q\,G\,a\,b\,2\,M\,457\,A}^{m\,u\,t-w\,t}$$

**FIGURE 5:**
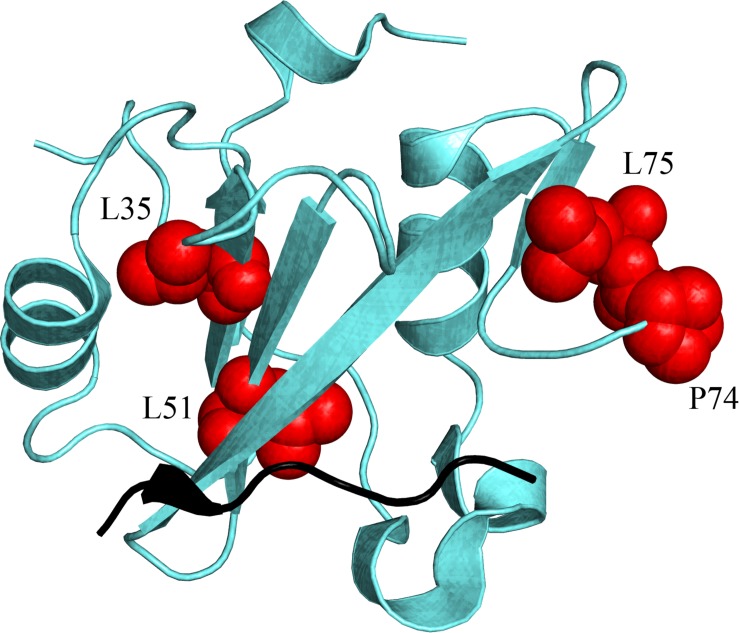
Structural distribution of the residues (highlighted in red on N-SH2 structure) that are energetically coupled with M residue part of the pY-X-X-M consensus that is specifically recognized by N-SH2, and finely modulate the affinity of the domain for its natural binding partners (see text for details). Because of the unavailability of the N-SH2:Gab2 complex structure, a different ligand is represented in black (cKit) only to pinpoint the position of the binding pocket of N-SH2. (PDB: 2IUH).

When ΔΔΔG = 0 the (de)stabilization of the bound state due to the point mutation in N-SH2 when binding to Gab2_448__–__460_ equals the one occurring in the binding with Gab2_448__–__460_ M457A, meaning that M457 is not energetically coupled to the mutated residue in N-SH2. On the contrary, a ΔΔΔG ≠ 0 implies that the two positions interact energetically. The energetic couplings for each mutated residue of N-SH2 with M457 of Gab2 were calculated from kinetic parameters obtained from the analysis of pseudo-first order binding experiments and are reported in Table 1. We found that 4 residues (L51, I60, L75, and P74) reported a ΔΔΔG > 0.4 kcal mol^–1^ upon binding with Gab2 M457A, whilst for residues L35, T48, L59, L99 we could not detect binding. It is of particular interest to analyse the structural distribution of the residues that are affected by M457A mutation (Figure 5). In fact, while T48, L59, I60, and L99 are located in the binding site, L35, L51, P74, L75 are far from the binding pocket. These results, under the light of NMR data and LFER analysis, evidence the presence of an allosteric network in the N-SH2 domain that involve the whole domain, suggesting a key role for these specific residues in modulating the recognition of M457 of Gab2 and the affinity for the substrate.

## Discussion

In spite of their importance for cell physiology, there is lack of information pertaining the details of the mechanism of binding of SH2 domains. In this paper, we provided a detailed experimental characterization of the binding of N-SH2 to one of its physiological ligands and detected an unexpected structural plasticity. This effect was monitored both directly, by NMR by comparing the free structure with those of the bound states with two different ligands, and indirectly, by analyzing the effects of mutagenesis on the binding capability of the domain. NMR data reported subtle conformational changes in free and bound states. These minor changes in chemical shifts, supported by LFER and ΔΔΔG analysis, are indicative of a fine allosteric regulation of the binding mechanism.

It is of interest to compare our findings with those obtained on the SH2 domain from Itk. In fact, in the latter, it was demonstrated binding to be regulated by peptidil-prolyl cis-trans isomerization (Mallis et al., 2002; Breheny et al., 2003; Pletneva et al., 2006; Severin et al., 2009). These findings appear to contrast the results reported in our work, which suggests that N-SH2 is characterized by a malleable structure, with binding being mediated by a diffused network involving several residues not directly located in the binding pocket, as mirrored by the linearity of the LFER plot. Remarkably, in stark contrast to what observed in the case of the SH2 domain from Itk, a quantitative analysis of our NMR data revealed no evidence for peptidil-prolyl cis-trans isomerization upon binding; therefore highlighting a completely different scenario. However, it is important to observe that P74 appears to retain a role in the recognition of M457 of Gab2, reporting a ΔΔΔG = 0.79 ± 0.12 kcal mol^–1^. Thus, this residue, while maintaining a cis conformation both in the free and bound states, nevertheless participates to the long range energetic network modulating binding. Interestingly, P74 is conserved in both N-SH2 and C-SH2 domains of PI3K, that are both known to recognize the pY-X-X-M consensus sequence (Dornan and Burke, 2018).

Long allosteric networks regulating binding reactions have been observed for different adaptor protein domains, such as PDZ and SH3 domains (Gianni et al., 2011; Malagrinò et al., 2019). Taken together with the experiments reported in this work, it appears that this mechanism of regulation may be typical of protein families that share the same topology but nevertheless must bind selectively specific ligands in the complex cellular environment. It has been demonstrated that SH2 domains can bind several peptides presenting different aminoacidic sequences containing a phosphotyrosine, with affinities differing of some orders of magnitude (Ladbury and Arold, 2000). We suggest that the conformational plasticity highlighted in our work, mediated by long range interactions, represents an additional mechanism of regulation of the SH2 moiety, tuning the selectivity of the domain, while maintaining a rather conserved topology and binding pocket. Future work on other SH2 domains will provide additional information about the generality of the allosteric regulation of the binding properties of SH2 domain family.

## Data Availability Statement

All datasets generated for this study are included in article/supplementary material.

## Author Contributions

LV, AD, and SG designed the research. LV, AT, JJ, FM, and FT performed the research. LV and AT wrote the first version of the manuscript. All the authors analyzed data and revised the manuscript.

## Conflict of Interest

The authors declare that the research was conducted in the absence of any commercial or financial relationships that could be construed as a potential conflict of interest.
